# Identifying Information Gaps in Electronic Health Records by Using Natural Language Processing: Gynecologic Surgery History Identification

**DOI:** 10.2196/29015

**Published:** 2022-01-28

**Authors:** Sungrim Moon, Luke A Carlson, Ethan D Moser, Bhavani Singh Agnikula Kshatriya, Carin Y Smith, Walter A Rocca, Liliana Gazzuola Rocca, Suzette J Bielinski, Hongfang Liu, Nicholas B Larson

**Affiliations:** 1 Department of Artificial Intelligence and Informatics Mayo Clinic Rochester, MN United States; 2 Division of Epidemiology, Department of Quantitative Health Sciences Mayo Clinic Rochester, MN United States; 3 Division of Clinical Trials and Biostatistics Department of Quantitative Health Sciences Mayo Clinic Rochester, MN United States; 4 Department of Neurology Mayo Clinic Rochester, MN United States; 5 Women’s Health Research Center Mayo Clinic Rochester, MN United States

**Keywords:** information gap, health information interoperability, natural language processing, electronic health records, gynecologic surgery, surgery, medical informatics, digital health, eHealth, gynecology

## Abstract

**Background:**

Electronic health records (EHRs) are a rich source of longitudinal patient data. However, missing information due to clinical care that predated the implementation of EHR system(s) or care that occurred at different medical institutions impedes complete ascertainment of a patient’s medical history.

**Objective:**

This study aimed to investigate information discrepancies and to quantify information gaps by comparing the gynecological surgical history extracted from an EHR of a single institution by using natural language processing (NLP) techniques with the manually curated surgical history information through chart review of records from multiple independent regional health care institutions.

**Methods:**

To facilitate high-throughput evaluation, we developed a rule-based NLP algorithm to detect gynecological surgery history from the unstructured narrative of the Mayo Clinic EHR. These results were compared to a gold standard cohort of 3870 women with gynecological surgery status adjudicated using the Rochester Epidemiology Project medical records–linkage system. We quantified and characterized the information gaps observed that led to misclassification of the surgical status.

**Results:**

The NLP algorithm achieved precision of 0.85, recall of 0.82, and F1-score of 0.83 in the test set (n=265) relative to outcomes abstracted from the Mayo EHR. This performance attenuated when directly compared to the gold standard (precision 0.79, recall 0.76, and F1-score 0.76), with the majority of misclassifications being false negatives in nature. We then applied the algorithm to the remaining patients (n=3340) and identified 2 types of information gaps through error analysis. First, 6% (199/3340) of women in this study had no recorded surgery information or partial information in the EHR. Second, 4.3% (144/3340) of women had inconsistent or inaccurate information within the clinical narrative owing to misinterpreted information, erroneous “copy and paste,” or incorrect information provided by patients. Additionally, the NLP algorithm misclassified the surgery status of 3.6% (121/3340) of women.

**Conclusions:**

Although NLP techniques were able to adequately recreate the gynecologic surgical status from the clinical narrative, missing or inaccurately reported and recorded information resulted in much of the misclassification observed. Therefore, alternative approaches to collect or curate surgical history are needed.

## Introduction

Electronic health records (EHRs) are a rich source of longitudinal patient information that can efficiently and cost-effectively be used for clinical care as well as for research. However, missing information due to clinical care that predated the implementation of EHR system(s) or that occurred at different medical institutions may result in an incomplete medical history. For example, gynecologic surgery history is essential for assessing women’s health, given the increased risk of aging-related outcomes among women undergoing these surgeries [1-6]. However, assessment of surgical status is complicated by the significant time interval (ie, decades) between these procedures and the subsequent aging-related events because these procedures occurred at different medical institutions. In addition, collecting comprehensive gynecological surgery history is challenging because various surgical combinations are performed: hysterectomy with or without oophorectomy, unilateral or bilateral oophorectomy, and unilateral oophorectomy followed by the removal of the remaining ovary at a later date.

The approaches to mitigate these types of information gaps in a patient’s medical history are (1) patient-provided information either by questionnaires or data collection during a clinical visit or (2) chart review. However, time constraints on providers can delay or prevent accurate assessment of medical history [7,8]. Further, patient-provided information can be limited or be inaccurate due to recall errors or lack of health literacy [9,10]. Manual chart abstraction of past medical records can overcome these issues but is often labor-intensive and time-consuming.

Natural language processing (NLP) techniques may be used to automatically extract relevant clinical information in a high throughput fashion. However, the medical history information of a patient is often a mix of paper records and EHRs distributed over multiple systems within or across multiple health care institutions [11]. This can be due to the evolution of clinical documentation at a single health care institution or the involvement of multiple health care institutions over the lifespan of patients. In some instances when upgrading EHR systems, past records are not loaded and some data elements may be completely dropped owing to differences in the underlying data models between the 2 systems [12,13]. In addition, patients can move in and out of health care institutions over time owing to personal preference, insurance coverage, or the referral process [14].

In this study, we had a unique opportunity to quantify information gaps by comparing the historical gynecologic surgery information obtained from EHR data of a single institution by using NLP techniques with the surgical history information that was manually curated through chart review of records from multiple independent regional health care institutions.

## Methods

### Gold Standard Cohort

The Mayo Clinic Cohort Study for Oophorectomy and Aging-2 (MOA-2) consisted of 570 women who underwent unilateral oophorectomy and 1653 women who underwent bilateral oophorectomy in Olmsted County, Minnesota between 1988 and 2007 before the age of 50 years [5,15,16]. Bilateral oophorectomy was defined as the removal of both ovaries in the same surgery or as the removal of the remaining ovary if 2 separate unilateral oophorectomies were performed. Women were excluded if they had undergone natural menopause before the oophorectomy. Women were also excluded if the oophorectomy was performed as a treatment for ovarian cancer, for estrogen-sensitive cancer, or if they carried a high-risk genetic variant. Each woman was matched by age (+/- 1 year) to a population-based referent woman who had not undergone any oophorectomy (570 unilateral referent women) or bilateral oophorectomy (1653 bilateral referent women) as of the date of surgery (index date) [5,15,16].

All women were identified using the Rochester Epidemiology Project (REP) medical records–linkage system [17-20]. Each health care provider in Olmsted County, Minnesota, uses a unit (or dossier) medical record system whereby all data collected on an individual are assembled in one place. Through the REP, these health care providers have agreed to share their patient records for research studies approved by the Institutional Review Boards of Mayo Clinic and Olmsted Medical Center [17]. In 2017, the REP contained approximately 2.3 million patient records from 54 different health care providers that matched to more than 591,000 individuals who had been residents of the Olmsted County at some point between 1966 and 2017. The REP captures virtually the entire population of Olmsted County as compared to the US Census (>99.9% of the 1970-2010 census counts) [18].

In MOA-2, available paper medical records and EHR data for each of the women were manually abstracted to confirm gynecological surgeries from all available REP sources before the index date and up to the last follow-up date. Thus, MOA-2 represents a gold standard data set with complete capture of surgical histories from all REP sources. Gynecological surgery status was divided into the following 6 mutually exclusive categories: bilateral oophorectomy only, hysterectomy and bilateral oophorectomy, unilateral oophorectomy only, hysterectomy and unilateral oophorectomy, hysterectomy only, and no surgery. Since each woman may have undergone multiple gynecological surgeries throughout her life (eg, an initial hysterectomy followed by a bilateral oophorectomy at a later date), a single status was assigned as of the latest individual follow-up date for each woman. Follow-up dates ranged from January 1997 through August 2019.

MOA-2 included 4446 women, of whom 173 were represented in the cohort twice, leaving 4273 unique women. For this study, we excluded women who died prior to the start of the Mayo Clinic EHR in 1997 (n=13), women without a Mayo Clinic medical record number (n=28), women who did not provide research authorization for medical records review (n=102), or women with no information available in the Mayo Clinic EHR (n=260). The final cohort consisted of 3870 unique women.

### Single-Institution Surgery Status Abstraction

Using labels from the gold standard, we randomly selected 100 women from each surgical status category for train and test sets (Table 1). However, owing to the rarity of “bilateral oophorectomy without hysterectomy,” only 30 women were included. The surgical status was then reviewed for women included in the train and test sets (n=530) by one of the 2 trained annotators (EDM and Ellen E Koepsell) using only data available within the Mayo EHR. The annotators were blinded to both the external gold standard status and abstractions of the other annotator. A stratified random sample by surgery type of 10.2% (54/530) of the women was additionally used to assess interannotator reliability, which was evaluated by percentage agreement and Cohen kappa.

**Table 1:** Gynecological surgical status of the patients in this study (N=3870).

Surgery status in MOA-2^a^	Train set (n=265)	Test set (n=265)	Remaining set (n=3340)
No surgery (n=1473)	50	50	1373
Bilateral oophorectomy only (n=35)	15	15	5
Hysterectomy and bilateral oophorectomy (n=1685)	50	50	1585
Unilateral oophorectomy only (n=214)	50	50	114
Hysterectomy and unilateral oophorectomy (n=247)	50	50	147
Hysterectomy only (n=216)	50	50	116

^a^MOA-2: Mayo Clinic Cohort Study of Oophorectomy and Aging-2.

### NLP

**Figure 1:**
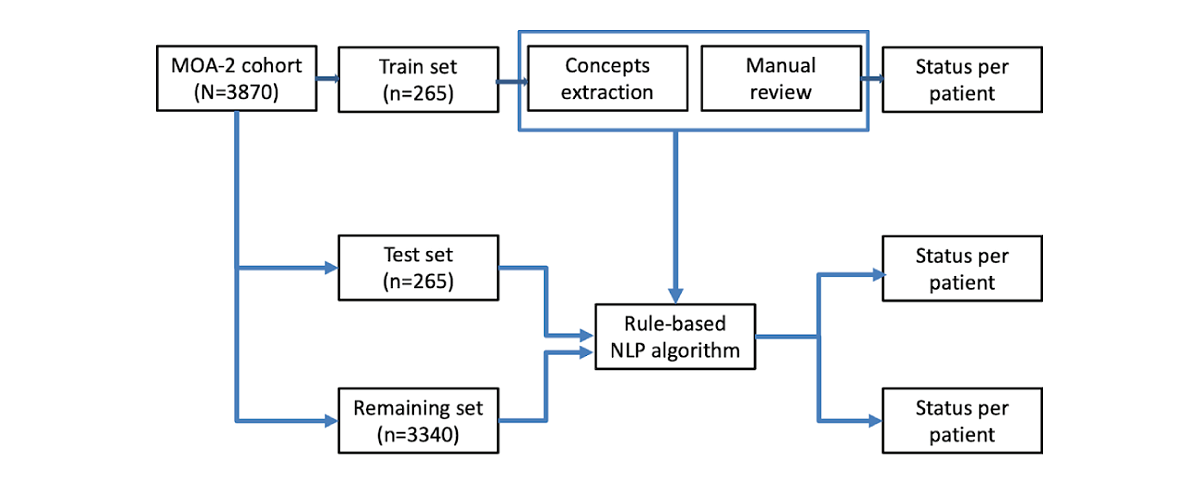
An overview of this study to classify the surgical histories of patients. MOA-2: Mayo Clinic Cohort Study of Oophorectomy and Aging-2; NLP: natural language processing.

To facilitate high-throughput surgical status extraction from the Mayo Clinic EHR, the train set was used to develop a rule-based NLP algorithm, and the test set was used to evaluate the NLP algorithm performance (Figure 1). The NLP algorithm was built using MedTagger applied to text from clinical notes, as well as pathology, radiology, and surgical operative reports in the Mayo Clinic EHR. MedTagger is a pipeline tool capable of extracting clinical events from the unstructured text given a clinical dictionary and ruleset [21]. MedTagger was designated as an NLP platform by Mayo Clinic for clinical NLP research. To develop the NLP algorithm to determine the status of gynecological surgery for each woman, MedTagger was adapted to extract surgery concepts within the clinical sections relevant to medical history and current clinical care (Multimedia Appendix 1). In detail, we utilized the series of the pipeline of MedTagger, such as sentence detection, tokenization, concept identification, and assertion. We aggregated the extracted concepts based on rules (Multimedia Appendix 2) at the patient level to determine the status of the patient’s surgery. For example, a sentence in clinical notes, “A total abdominal hysterectomy with bilateral salpingo-oophorectomy was performed the usual fashion,” triggers 2 concepts, “hysterectomy” and “bilateral salpingo-oophorectomy,” through the pipeline of MedTagger. The NLP algorithm determines the patient’s surgery status as “Hysterectomy and bilateral oophorectomy." Only concepts relevant to the women (ie, not family history) with positive and assertive contextual information were considered valid. If the sentence included a valid oophorectomy concept and contained the word “left,” this was categorized as “left side oophorectomy,” whereas those having the word “right” were categorized as “right side oophorectomy.” During the process of aggregating the extracted concepts on the patient level, if none of the concepts contain the laterality of a unilateral oophorectomy surgery, it was considered “left side oophorectomy” as default and classified as unilateral oophorectomy. The final surgical status for each woman was determined by applying rules to all valid concepts relevant to the woman (Multimedia Appendix 2). Because temporal information is also critical, we explored the extraction of the surgery date information for 3 types of surgeries, that is, unilateral oophorectomy, bilateral oophorectomy, and hysterectomy in the train and test sets (n=530). We extracted all date information based on 3 patterns, that is, DD/MM/YYYY, DD/MM/YY, or YYYY from sentences containing the surgery information.

### Performance Evaluation

To evaluate the performance of the NLP algorithm, we calculated precision (ie, positive predictive value), recall (ie, sensitivity), F1-score, and accuracy. Precision represented the proportion of women that the NLP algorithm determined as having surgery who truly had the surgery. Recall indicated the proportion of women who truly had surgery and were determined by the NLP algorithm as having had surgery. F1-score was the harmonized measurement between precision and recall. Accuracy was the proportion of correctly classified surgery statuses by the NLP algorithm. All performance measures were calculated both with respect to surgical status ascertained from the Mayo Clinic EHR as well as the MOA-2 gold standard. Since we have a limited number of women with bilateral oophorectomy only, we reported both macro average metrics for overall surgery status (which calculated the matrix independently by surgery status but not considering weights for sample size) and weighted average metrics for overall surgery status (with weighting by sample size). Recognizing that missing data are common owing to movement in and out of health care systems, we also analyzed the recovery ratio of the surgery status information (using the weighted average F1-score) between the limited and reverse-chronological years of records and the total years of records by the NLP algorithm.

### Discrepancy Analysis

After training and validating the NLP algorithm, it was subsequently applied to all remaining Mayo records. All discrepancies between NLP classifications and gold standard MOA-2 data were then identified and manually reviewed by 1 annotator (EDM), which were subsequently classified into 3 categories: external information gaps, internal information gaps, and technical errors by the NLP algorithm. External discrepancies were defined as differences in surgical status between the 2 sources (eg, the gold standard categorizes a woman as having surgery, but the surgery is not mentioned in the Mayo Clinic EHR) and were reviewed by another annotator (LGR, a physician) to determine the true surgical status. Internal discrepancies were differences due to inconsistent or inaccurate surgery history information in the Mayo Clinic EHR (eg, partial vs complete surgery). Finally, we also identified technical errors by the NLP algorithm (eg, negated but classified as positive).

### Ethics Approval

The study was approved by the Mayo Clinic and Olmsted Medical Center Institutional Review Boards.

## Results

### Corpus Analysis and Results of the NLP Algorithm on the Train and Test Sets

In this cohort, the median age at follow-up was 60 years (IQR 54-66 years), and the median length of follow-up was 16.2 years (IQR 11.1-21.1 years). Among 3870 women, 1473 (38.1%) did not undergo gynecologic surgery, while 2397 (61.9%) underwent at least one gynecologic surgery before their latest follow-up date. Most women with gynecologic surgery history (2069/2397, 86.3%) had only 1 surgical date, 12.7% (304/2397) had 2 separate surgery dates, and 1% (24/2397) had 3 separate surgery dates.

Among the 54 cases selected for interannotator reliability assessment, the percentage agreement was 90.7% (49/54) and the kappa statistic was 0.85. Of the 530 patients initially selected for annotation, 446 (84.2%) were accurately annotated using the Mayo EHR compared to the MOA-2 gold standard (Multimedia Appendix 3). In general, disagreement between Mayo-annotated and MOA-2 gynecologic surgery statuses was large with respect to false negatives (ie, Mayo annotations inaccurately assigned to “no surgery”), which comprised 59 of the 84 total misclassifications (70.2%).

We present the test-set performance metrics relative to the Mayo annotation labels and MOA-2 gold standard labels in Table 2 (and train set performances reported in Multimedia Appendix 4). Using surgical statuses extracted from the Mayo EHR, the NLP algorithm correctly classified 82.3% of women (218/265 women in the test set), with weighted averages of 0.85 precision, 0.82 recall, and 0.83 F1-score. When compared to the MOA-2 labels, performance dropped moderately and the surgical status of 76.2% of the women (202/265 women in the test set) was correctly classified by the NLP algorithm through the follow-up date. The NLP algorithm achieved precision of 0.79, recall of 0.76, and weighted average F1-score of 0.76 in the test set (Table 2). Performance measures varied by the surgery type, with the lowest performance observed for assessing “bilateral oophorectomy only” and the highest for identifying “hysterectomy only.”

**Table 2:** Test set evaluation (n=265) of the natural language processing algorithm using Mayo and Mayo Clinic Cohort Study of Oophorectomy and Aging-2 annotations.

Algorithm surgery type	Mayo				MOA-2^a^		
	Precision	Recall	F1-score	Precision		Recall	F1-score
No surgery	0.98	0.81	0.89	0.74		0.96	0.83
Bilateral oophorectomy only	0.42	0.62	0.50	0.58		0.47	0.52
Hysterectomy and bilateral oophorectomy	0.68	1.00	0.81	0.62		0.90	0.73
Unilateral oophorectomy only	0.94	0.74	0.83	0.97		0.66	0.79
Hysterectomy and unilateral oophorectomy	0.84	0.71	0.77	0.84		0.64	0.73
Hysterectomy only	0.81	0.88	0.84	0.86		0.74	0.80
Overall macro average	0.78	0.79	0.77	0.77		0.73	0.73
Overall weighted average	0.85	0.82	0.83	0.79		0.76	0.76

^a^MOA-2: Mayo Clinic Cohort Study of Oophorectomy and Aging-2.

**Figure 2:**
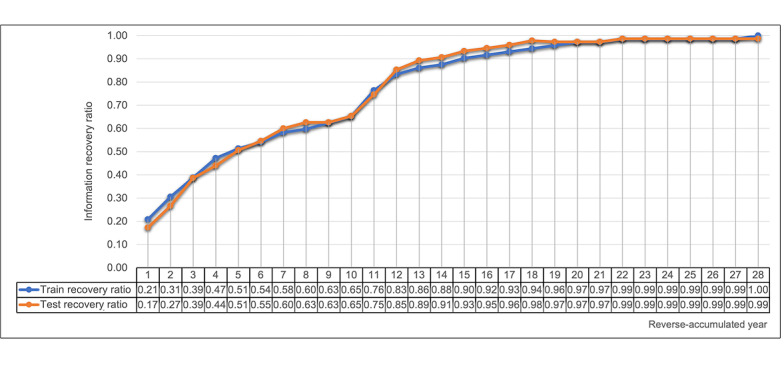
Recovery ratio for the surgery status information by years of electronic health record data available.

If we restricted the NLP algorithm to use recent clinic notes in the reverse-chronological order from individual follow-up dates, 1 year of clinical notes yielded only 17.1% (0.13/0.76) of the surgical status information compared to the original weighted average F1-score of 0.76. A minimum of 14 years of narrative notes in the test set was required for the NLP algorithm to recover 90% of the surgical status information. The overall trend of the weighted average F1-score recovery ratio according to reverse-chronological year is represented in Figure 2. About 62.3% (268/430) of women had the surgery date information present in at least one clinical note. We also observed a disparity in date information by surgery status. Specifically, only 23% (46/200) of women with unilateral oophorectomy surgery had the date information present. In contrast, 70% (91/130) of women with bilateral oophorectomy and 82.7% (248/300) of women with hysterectomy had the date information present.

### Results of the NLP Algorithm on the Remaining Set and Discrepancy Analysis

When we applied the NLP algorithm to the remaining set (n=3340), we correctly classified 86.1% (2876/3340) of the surgery status of patients. Similar to the test set results, recall rates were relatively poor for positive surgical history. In Table 3, we summarized 464 discrepancies of surgical status in the NLP algorithm classification compared to the multi-institutional MOA-2 gold standard. First, 6% (199/3340) of women in this set had either no recorded surgery information or partial information in the EHR. Second, we found inconsistent or inaccurate information for 4.3% (144/3340) of women. Lastly, the NLP algorithm misclassified the surgery status of 3.6% (121/3340) of women. External information gaps represented 42.9% (199/464) of the discrepancies, internal information gaps represented 31% (144/464) of the discrepancies, and 26.1% (121/464) were technical errors of the NLP algorithm.

**Table 3:** Summary of the 464 discrepancies observed.

Type, categorization			Value (n)
**External information gap**			
	**Mayo electronic health record**		
		Missing information	92
		Partial information	49
	**Gold standard**		
		Missing information	11
		Partial information	45
	**Both**		
		Partial information	2
**Internal information gap**			
	**Correction over time**		
		Documented surgeries but revealed later as no surgeries	74
	**Irregular concept scope**		
		Partial surgery versus complete surgery	17
		Biopsy examination versus complete surgery	23
		Planned surgery versus real surgery	5
	**Miscommunication within clinical documents**		
		Hysterectomy versus hysteroscopy	8
		Incorrect laterality (left vs right-side) information	12
		Typo	5
**Technical errors of natural language processing**			
	**Complicated context**		
		Discussion versus real surgery	63
		Family history versus history of patient	11
		Complex expressions of partial surgery	6
		Complex expressions of laterality information	12
	**Incorrect certainty**		
		Negated but classified as positive	10
		Positive but classified as negated	1
		Positive but classified as hypothetical	1
	**Unknown features**		
		Irrelevant section header	2
		Unknown keywords/rules	15

Of the 199 external information gaps, positive surgical history was missing in the Mayo Clinic EHR for 92 women (ie, false negatives, 46.2%). In contrast, the surgical history present in the Mayo Clinic EHR was not captured by the gold standard for 11 women. There were discrepancies related to surgery type for 96 women. The details for all external information gaps are summarized in Multimedia Appendix 5.

Of the 144 internal information gaps identified, the chart review revealed multiple potential sources of inconsistency. The details regarding the surgery type were frequently inconsistent, and about half of the discrepancies (n=74) resulted from the correction of surgery information over time. For example, one note for a patient indicated “BSO” (bilateral salpingo-oophorectomy), whereas all other notes contained “remained right ovary,” indicating a unilateral oophorectomy. There were differences between clinical notes and the more detailed surgical or pathology reports (n=45). Miscommunication within clinical documents in the use of words (eg, misinterpretation of “hysteroscopy” noted as “hysterectomy”), incorrect laterality (ie, left vs right), and typos were also observed (n=25).

Finally, there were 121 technical errors in the NLP algorithm. The NLP algorithm had difficulties in accurately processing complicated contextual information (n=92). For example, it had difficulty distinguishing discussion/consideration from real surgery or patient history from family history. In addition, the NLP algorithm misclassified certainty information of sentences (eg, negated but classified as positive, n=12), or it missed the surgical information owing to the limited set of keywords/phrases or associated section header information (n=17). For example, a subtitle in the surgery operative notes, “Uterus, endometrium, hysterectomy: Inactive” was classified as a valid “hysterectomy” by the NLP algorithm.

## Discussion

A comprehensive medical history of individual patients is necessary to achieve a high quality of patient care and to support clinical research. Identifying historical surgery information is challenging because some surgeries may have occurred decades before the widespread adoption of EHR systems. Furthermore, useful information is often distributed in separate EHR systems owing to the preference or needs of the patients. Finally, limited time during clinic visits and quality of self-reported history often result in incomprehensive surgery information. This study sought to extract gynecological surgical history from a single EHR by using a rule-based NLP algorithm and to compare these results with gold standard data ascertained from a manual multi-institutional record review.

The NLP algorithm that was trained on surgery statuses manually extracted from the Mayo EHR was largely successful with respect to being internally valid; however, false negatives were commonly encountered when compared to gold standard information. In addition to misclassification, the date of the surgery was often missing, rendering ascertainment of surgery timing difficult. The preponderance of false negatives is consistent with a model of information loss over the lifetime of a patient and may serve as a source of systematic bias in research.

The external information gaps were the most common errors encountered and related to missing or incomplete information in the EHR for surgery status or surgery type. Similar to the test set results, we observed that nearly 50% of the external discrepancies were false negative in nature. These results starkly contrast diagnostic code–based results reported by Rocca et al [16] when using the full resources of the REP to build the MOA-2 cohort, which were highly accurate in identifying surgical history status for oophorectomy. In addition, the longer a woman was followed in the EHR, the more likely her gynecologic surgery was recorded in the clinical narrative. This is again fairly intuitive, as follow-up time within a single EHR system likely captures consistent and reliable information with fewer opportunities for data loss in record transfers. Surgical date information was sparse and differed by surgery type. For example, patients commonly provided their age at the time of surgery rather than the surgery date. Consequently, research that relies on reliable ascertainment of surgery dates should take these heterogeneous and complex modes of information representation into consideration. The most common internal information gap identified was inaccurate reporting of surgical status by the clinician, the patient, or both that was subsequently refuted. Thus, information conflict resolution is another critical element to address in information extraction from long-term clinical narratives.

With the growing popularity of utilizing NLP-based phenotyping for research using EHR data, it is important to consider the nonnegligible risk of misclassification despite evidence of internal validity for NLP-based phenotyping algorithms. Systematic misclassification toward false negatives could induce biases in research, particularly for patient populations that are highly transient and may change care providers frequently. Strategies to reduce information gaps and to improve the collection of surgical history include leveraging the NLP technology with optical character recognition technology to digitalize paper-based records or acquiring the records digitally via a health information exchange [22-25]. Lastly, the implementation of systematic questionnaires to gather prior surgical information may significantly reduce information gaps as well. The questionnaires can also be leveraged for capturing potential documentation errors besides enhancing documentation quality.

The strengths of our study include the total sample size available and the high-quality gold standard phenotype data. However, the performance of the simple rule-based NLP algorithm could be improved upon with more sophisticated methods, as indicated by the extent of technical errors identified in the discrepancy analysis.

In conclusion, our study demonstrated the overall feasibility of extracting gynecological surgeries that often predated the EHR system by decades using a rule-based NLP algorithm. However, we identified external and internal information gaps by comparing NLP algorithm results to a manually abstracted gold standard. Additional efforts are necessary to mitigate these information gaps and include the use of advanced NLP techniques to process paper medical records and systematic collection and documentation of surgical history.

## Appendix

Multimedia Appendix 1 Commonly used keywords and phrases to describe gynecologic surgeries and associated clinical note section headers used in the algorithm.

Multimedia Appendix 2 Rule to determine final gynecologic surgery status.

Multimedia Appendix 3 Annotation comparison between Mayo and Mayo Clinic Cohort Study of Oophorectomy and Aging-2 gold standard.

Multimedia Appendix 4 Training set (n=265) evaluation of the natural language processing algorithm using Mayo and Mayo Clinic Cohort Study of Oophorectomy and Aging-2 annotations.

Multimedia Appendix 5 Distribution of the 199 external information discrepancies observed.

## Abbreviations

EHR: electronic health record
MOA-2: Mayo Clinic Cohort Study for Oophorectomy and Aging-2
NLP: natural language processing
REP: Rochester Epidemiology Project
