# Supplementary material for: Identifying Information Gaps in Electronic Health Records by Using Natural Language Processing: Gynecologic Surgery History Identification
Source: J Med Internet Res. 2022 Jan 28;24(1):e29015. doi: 10.2196/29015 (PMC8838563; doi:10.2196/29015)
Supplement: Multimedia Appendix 1 [file jmir_v24i1e29015_app1.docx]

**Table S1.** Commonly used keywords and phrases to describe gynecologic surgeries and associated clinical note section headers used in the algorithm.

| Gynecologic surgery concepts | Keywords/phrases |
| --- | --- |
| Broad concept of oophorectomy | salpingo oophorectomy  ovariectomy |
| Unilateral oophorectomy | (unilateral\|first) salpingo  (oophorectomy\|ovariectomy)  remov(ed\|al) ovary  ovary remov(ed\|al) |
| Bilateral oophorectomy | (bilateral\|second) salpingo (oophorectomy\|ovariectomy)  remov(ed\|al) ovaries  ovaries remov(ed\|al)  BSO^a^ |
| Hysterectomy | total (vaginal\|abdominal) hysterectomy  (vag\|abd) hyst  TVH^b^, TAH^c^ |
| Clinical Note Section Headers | Revision History  History of Present Illness  Past Medical/Surgical History  Impression/Report/Plan  Procedures |

^a^BSO: Bilateral Salpingo Hysterectomy.

^b^TAH: Total Abdominal Hysterectomy.

^c^TVH: Total Vaginal Hysterectomy.
