# Supplementary material for: Identifying Information Gaps in Electronic Health Records by Using Natural Language Processing: Gynecologic Surgery History Identification
Source: J Med Internet Res. 2022 Jan 28;24(1):e29015. doi: 10.2196/29015 (PMC8838563; doi:10.2196/29015)
Supplement: Multimedia Appendix 2 [file jmir_v24i1e29015_app2.docx]

**Table S2.** Rule to determine final gynecologic surgery status.

| Surgical Status | Left side  oophorectomy | Right side  oophorectomy | Unilateral  oophorectomy | Bilateral  oophorectomy | Hysterectomy |
| --- | --- | --- | --- | --- | --- |
| No surgery | No | No | No | No | No |
| Bilateral oophorectomy only | Yes or No | Yes or No | Yes or No | Yes | No |
|  | Yes | Yes | Yes or No | No | No |
| Hysterectomy and bilateral oophorectomy | Yes or No | Yes or No | Yes or No | Yes | Yes |
|  | Yes | Yes | Yes or No | No | Yes |
| Unilateral oophorectomy only | No | No | Yes | No | No |
|  | Yes | No | Yes or No | No | No |
|  | No | Yes | Yes or No | No | No |
| Hysterectomy and unilateral oophorectomy | No | No | Yes | No | Yes |
|  | Yes | No | Yes or No | No | Yes |
|  | No | Yes | Yes or No | No | Yes |
| Hysterectomy only | No | No | No | No | Yes |
