# Supplementary material for: Identifying Information Gaps in Electronic Health Records by Using Natural Language Processing: Gynecologic Surgery History Identification
Source: J Med Internet Res. 2022 Jan 28;24(1):e29015. doi: 10.2196/29015 (PMC8838563; doi:10.2196/29015)
Supplement: Multimedia Appendix 3 [file jmir_v24i1e29015_app3.docx]

**Table S3.** Annotation comparison between Mayo and Mayo Clinic Cohort Study of Oophorectomy and Aging-2 gold standard.

|  | Mayo electronic health record annotations | | | | | |
| --- | --- | --- | --- | --- | --- | --- |
| MOA-2^a^ gold standard, *N* | No surgery (n=158) | Bilateral oophorectomy only (n=14) | Hysterectomy and bilateral oophorectomy (n=100) | Unilateral oophorectomy only (n=89) | Hysterectomy and unilateral oophorectomy (n=84) | Hysterectomy only (n=85) |
| No surgery (n=101) | 99 | 0 | 0 | 2 | 0 | 0 |
| Bilateral oophorectomy only (n=30) | 10 | 14 | 6 | 0 | 0 | 0 |
| Hysterectomy and bilateral oophorectomy (n=100) | 6 | 0 | 89 | 0 | 1 | 4 |
| Unilateral oophorectomy only (n=100) | 12 | 0 | 0 | 86 | 0 | 2 |
| Hysterectomy and unilateral oophorectomy^b^ (n=99) | 12 | 0 | 1 | 1 | 82 | 3 |
| Hysterectomy only (n=100) | 19 | 0 | 4 | 0 | 1 | 76 |

^a^MOA-2: Mayo Clinic Cohort Study of Oophorectomy and Aging-2.

^b^One “Hysterectomy and unilateral oophorectomy case” of original the gold standard of MOA-2 was fixed the status as “No surgery” case after natural language processing results were compared to the gold standard of MOA-2 and extensive manual review processing with several resources.
