# Supplementary material for: Identifying Information Gaps in Electronic Health Records by Using Natural Language Processing: Gynecologic Surgery History Identification
Source: J Med Internet Res. 2022 Jan 28;24(1):e29015. doi: 10.2196/29015 (PMC8838563; doi:10.2196/29015)
Supplement: Multimedia Appendix 4 [file jmir_v24i1e29015_app4.docx]

**Table S4.** Training set (n=265) evaluation of the natural language processing algorithm using Mayo and Mayo Clinic Cohort Study of Oophorectomy and Aging-2 annotations.

| Algorithm surgery type | Mayo | | | MOA-2^a^ | | |
| --- | --- | --- | --- | --- | --- | --- |
|  | Precision | Recall | F1-score | Precision | Recall | F1-score |
| No surgery | 1.00 | 0.81 | 0.90 | 0.78 | 0.98 | 0.87 |
| Bilateral oophorectomy only | 0.42 | 0.83 | 0.56 | 0.58 | 0.47 | 0.52 |
| Hysterectomy and bilateral oophorectomy | 0.63 | 1.00 | 0.78 | 0.57 | 0.90 | 0.70 |
| Unilateral oophorectomy only | 0.97 | 0.80 | 0.88 | 1.00 | 0.76 | 0.86 |
| Hysterectomy and unilateral oophorectomy | 0.83 | 0.62 | 0.71 | 0.83 | 0.49 | 0.62 |
| Hysterectomy only | 0.81 | 0.78 | 0.80 | 0.86 | 0.74 | 0.80 |
| Overall accuracy | 0.81 | | | 0.76 | | |
| Overall macro average | 0.78 | 0.81 | 0.77 | 0.77 | 0.72 | 0.73 |
| Overall weighted average | 0.86 | 0.81 | 0.82 | 0.79 | 0.76 | 0.76 |

^a^MOA-2: Mayo Clinic Cohort Study of Oophorectomy and Aging-2.
