# Supplementary material for: Identifying Information Gaps in Electronic Health Records by Using Natural Language Processing: Gynecologic Surgery History Identification
Source: J Med Internet Res. 2022 Jan 28;24(1):e29015. doi: 10.2196/29015 (PMC8838563; doi:10.2196/29015)
Supplement: Multimedia Appendix 5 [file jmir_v24i1e29015_app5.docx]

**Table S5.** Distribution of the 199 external information discrepancies observed.

| MOA-2^a^ | Mayo electronic health record | *N* |
| --- | --- | --- |
| No surgery | Bilateral oophorectomy only | 2 |
| No surgery | Hysterectomy and bilateral oophorectomy | 1 |
| No surgery | Unilateral oophorectomy only | 6 |
| No surgery | Hysterectomy only | 2 |
| Bilateral oophorectomy only | No surgery | 1 |
| Bilateral oophorectomy only | Hysterectomy and bilateral oophorectomy | 1 |
| Bilateral oophorectomy only | Hysterectomy only | 1 |
| Hysterectomy and bilateral oophorectomy | No surgery | 33 |
| Hysterectomy and bilateral oophorectomy | Bilateral oophorectomy only | 1 |
| Hysterectomy and bilateral oophorectomy | Unilateral oophorectomy only | 1 |
| Hysterectomy and bilateral oophorectomy | Hysterectomy and unilateral oophorectomy | 8 |
| Hysterectomy and bilateral oophorectomy | Hysterectomy only | 31 |
| Unilateral oophorectomy only | No surgery | 22 |
| Unilateral oophorectomy only | Bilateral oophorectomy only | 3 |
| Unilateral oophorectomy only | Hysterectomy only | 1 |
| Hysterectomy and unilateral oophorectomy | No surgery | 20 |
| Hysterectomy and unilateral oophorectomy | Hysterectomy and bilateral oophorectomy | 32 |
| Hysterectomy and unilateral oophorectomy | Hysterectomy only | 8 |
| Hysterectomy only | No surgery | 16 |
| Hysterectomy only | Hysterectomy and bilateral oophorectomy | 3 |
| Hysterectomy only | Hysterectomy and unilateral oophorectomy | 6 |

^a^MOA-2: Mayo Clinic Cohort Study of Oophorectomy and Aging-2.
